# Diuretic prescriptions in the first year of haemodialysis: international practice patterns and associations with outcomes

**DOI:** 10.1093/ckj/sfae141

**Published:** 2024-06-14

**Authors:** Nahid Tabibzadeh, Dongyu Wang, Angelo Karaboyas, Elke Schaeffner, Stefan H Jacobson, Almudena Vega, Kosaku Nitta, Brian Bieber, Roberto Pecoits-Filho, Pablo Antonio Ureña Torres

**Affiliations:** Nephrology Division, Massachusetts General Hospital, Boston, MA, USA; Arbor Research Collaborative for Health, Ann Arbor, MI, USA; Arbor Research Collaborative for Health, Ann Arbor, MI, USA; Department of Nephrology, Institute of Public Health, Charité-Universitätsmedizin Berlin, Berlin, Germany; Division of Nephrology, Department of Clinical Sciences, Karolinska Institutet, Danderyd University Hospital, Stockholm, Sweden; Nephrology Department, Hospital General Universitario Gregorio Marañón, Madrid, Spain; Department of Nephrology, Tokyo Women's Medical University, Shinjuku, Tokyo, Japan; Arbor Research Collaborative for Health, Ann Arbor, MI, USA; Arbor Research Collaborative for Health, Ann Arbor, MI, USA; Department of Dialysis, AURA Nord Saint Ouen, Saint-Ouen, France; Department of Renal Physiology, Necker Hospital, University of Paris Descartes, Paris, France

**Keywords:** diuretics, DOPPS, haemodialysis

## Abstract

**Background:**

The use of diuretics in patients on haemodialysis (HD) is thought to maintain diuresis. However, this assumption and the optimal dose are based on little scientific evidence, and associations with clinical outcomes are unclear.

**Methods:**

We reported international variations in diuretic use and loop diuretic dose across 27 759 HD patients with dialysis vintage <1 year in the Dialysis Outcomes and Practice Patterns Study phases 2–5 (2002–2015), a prospective cohort study. Doses of torsemide (4:1) and bumetanide (80:1) were converted to oral furosemide-equivalent doses. Adjusted Cox, logistic and linear regressions were used to investigate the association of diuretic use and dose with outcomes.

**Results:**

Diuretic utilization varied widely by country at vintage <3 months, ranging from >80% in Germany and Sweden to <35% in the USA, at a median dose ranging from 400–500 mg/day in Germany and Sweden to <100 mg/day in Japan and the USA. Neither diuretic use nor higher doses were associated with a lower risk of all-cause mortality, a higher risk of hospitalization for fracture or elevated parathyroid hormone levels, but the prescription of higher doses (>200 mg/day) was associated with a higher risk of all-cause hospitalization.

**Conclusions:**

Substantial international differences exist in diuretic prescriptions, with use and doses much higher in some European countries than the USA. The prescription and higher doses of loop diuretics was not associated with improved outcomes.

KEY LEARNING POINTS **What was known:**

- There is a substantial variation in diuretic use worldwide in dialysis patients (30–80%).

**This study adds:**

- The average dose ranges from 50 to 500 mg/day across participating countries.

**Potential impact:**

- High doses of diuretic were not associated with improved outcomes.

## INTRODUCTION

The use of diuretics is highly prevalent in patients with chronic kidney disease (CKD) not yet on dialysis, as they offer several benefits, including better management of steady-state fluid balance, sodium and potassium accumulation and arterial blood pressure (BP) [1]. One of the two most used diuretic drugs are loop diuretics, which inhibit sodium–potassium–chloride co-transporter 2 of the thick ascending limb of the loop of Henle.

While loop diuretics do not appear as a first-line therapy in hypertension guidelines for the general population [2], the 2004 Kidney Disease Outcomes Quality Initiative guidelines recommend their use in CKD stages 4–5, at a daily dose of 40–80 mg of furosemide to treat CKD-associated hypertension [3]. In the same line, the 2012 Kidney Disease: Improving Global Outcomes guidelines consistently recommend them in patients with an estimated glomerular filtration rate (eGFR) <30 ml/min/1.73 m^2^ within a combination therapy, especially in patients with sodium retention and a patent oedema state [4].

In contrast, no precise guidelines exist in incident and prevalent haemodialysis (HD) patients, although loop diuretics are routinely administered to patients. Besides their recognized beneficial effects during CKD, they may allow a longer preservation of residual urinary volume (RUV) and better protection from the consequences of volume overload and hyperkalaemia in these patients [5-7], outcomes that are associated with improved survival and quality of life [8, 12]. Indeed, they effectively increase urine volume output and sodium, chloride and potassium excretion in small as well as high doses, and in HD as well as peritoneal dialysis (PD) patients [13-15]. However, high doses are usually necessary to achieve sufficient loop exposure to the drug in patients with reduced GFR [16], yet high-dose formulations (i.e. 500 mg tablets of furosemide) are not available in the USA [17], thus limiting their use.

As a consequence or a cause, in clinical practice the use of diuretics varies significantly around the world, with largely routine use in Europe at HD initiation, compared with less frequent use in the USA [1, 18]. For instance, a study of United States Renal Data System data reported that the use of diuretics was ≈70% in the 3 months before dialysis initiation and decreased to ≈30% in the first 3 months after starting dialysis, which continued to decrease to 23% after 2 years [1]. Comparable results have also been reported from the Dialysis Outcome and Practice Patterns Study (DOPPS), which showed that within the first 90 days of starting HD, diuretic use markedly declined in the USA, Japan and Europe [19]. This study also showed that the use of diuretics was associated with a lower interdialytic weight gain (IDWG) and lower risk of hyperkalaemia and cardiovascular mortality [19].

A more recent study evaluating diuretics continuation after HD initiation, using Medicare electronic records in the USA, found that patients who continued diuretic treatment had a lower risk of hospitalization, less IDWG and less intradialytic hypotension, but no difference in mortality during a 1-year follow-up [20]. The study did not report on dosage regimens or on cardiovascular or mineral and bone disorder (MBD) outcomes, which are of interest since loop diuretics increase calciuria, hence potentially secondary hyperparathyroidism, and reduce bone mineral density [21, 22].

With the absence of robust data supporting the use of diuretics in HD patients, especially a high-dose regimen, and to help better inform international therapeutic strategies, we investigated the regional variation in diuretic use and dosing strategies as well as associations between diuretic prescription practices and outcomes, including mortality, hospitalization, MBD markers and extracellular volume parameters.

## MATERIALS AND METHODS

### Data source

The DOPPS is a prospective cohort study of adult chronic in-centre HD patients in 21 countries, ongoing since 1996. Maintenance HD patients were randomly selected from HD facilities in each country; detailed information is included in prior research [23, 24] and at http://www.dopps.org. Study approval and patient consent were obtained as required by national and local ethics committee regulations. The DOPPS includes data on patient demographics, socio-economic measures, comorbidities, laboratory measures, medication prescriptions and prospective follow-up for hospitalization and mortality.

### Study sample

In this analysis we utilized DOPPS data from phase 2 (2002–2005) through phase 5 (2012–2015). In exploratory analyses describing diuretic use by dialysis vintage, we used data from 76 395 HD patients. For the primary analyses investigating diuretic prescription practices in more detail, including international variations and associations with clinical outcomes, MBD markers and extracellular volume parameters, we restricted analyses to patients with a dialysis vintage of <1 year at DOPPS enrolment (Supplementary Fig. S1).

Descriptive analyses by dialysis vintage did not have a vintage restriction; all subsequent analyses were restricted to a vintage of <1 year to reflect a study population where HD patients are more likely candidates for diuretic prescription. Patients missing data on diuretic prescription were excluded; facilities with fewer than five HD patients with a vintage of <1 year were excluded from analyses because the ‘facility preference’ practice pattern could not be reliably defined.

### Variables

The primary exposure variable is diuretic use, defined as active prescription at patient enrolment in DOPPS. While diuretic prescription was captured periodically during follow-up (every 4 months), exact start/stop dates were unknown, so we chose to only utilize the baseline prescription for purposes of this analysis. The exposure was parameterized as any diuretic use (yes/no) and loop diuretic dose (0, 1–60, 61–200, >200 mg/day). Doses were based on the active prescription at the time of DOPPS enrolment and were not updated during follow-up. To avoid conversion issues, all analyses of diuretic doses were restricted to loop diuretics, which represent >90% of diuretic use in this study. Doses of torsemide (4:1) and bumetanide (80:1) were converted to oral furosemide-equivalent doses [25]. To help avoid confounding by indication bias due to differences between HD patients prescribed versus not prescribed a diuretic, we also explored secondary exposures using an HD facility preference approach [26], an approach utilized in prior analyses of the DOPPS data [27]. Each patient was assigned the exposure status of their HD facility, based on the proportion of patients prescribed a diuretic, facility mean loop diuretic dose among loop diuretic users and facility mean loop diuretic dose (including non-users as 0 dose).

Predefined outcomes were grouped into three categories: clinical outcomes, MBD markers and extracellular volume parameters. Time-to-event clinical outcomes include all-cause mortality, all-cause hospitalization, hospitalization due to major adverse cardiovascular events (MACE) or heart failure (HF), hospitalization due to HF and hospitalization due to fracture. MBD outcomes include serum calcium, serum phosphorus, parathyroid hormone (PTH) and total alkaline phosphatase (ALP). Extracellular volume outcomes include systolic BP (SBP) and diastolic BP (DBP) (average of three pre-dialysis measurements during 1 week), IDWG (average of three intervals over the course of 1 week: 1st to 2nd session, 2nd to 3rd session and 3rd to 1st session) and RUV (defined as urine output >200 ml/day).

### Statistical analysis

We first explored diuretic prescription patterns across categories of dialysis vintage by country and RUV. All subsequent analyses were restricted to patients with a dialysis vintage <1 year.

The proportions of patients prescribed a diuretic—overall and by type—are reported by country, region and DOPPS phase. The facility proportion of patients prescribed a diuretic is also reported by country. Loop diuretic dose is summarized at the patient level and facility level by country. Baseline patient characteristics are summarized by diuretic use, loop diuretic dose, facility percent diuretic use and facility mean loop diuretic dose.

We investigated the associations of patient-level diuretic prescription (including use and dose) with clinical outcomes, MBD markers and extracellular volume parameters. For time-to-event clinical outcomes, we used Cox regression to estimate hazard ratios (HRs) with 95% confidence intervals (CIs). Cox models were stratified by DOPPS phase and country, with a robust sandwich covariance estimator to account for facility clustering. Time at risk began at DOPPS enrolment and ended at the time of the event of interest, death, 7 days after leaving the facility due to transfer or change in modality, loss to follow-up or administrative end of the study phase (whichever occurred first). For continuous MBD laboratory outcomes, we used linear mixed models to estimate the difference in means (95% CIs). For continuous extracellular volume outcomes, we similarly used linear mixed models to estimate the difference in means (95% CIs). For binary extracellular volume outcomes (i.e. RUV), we used logistic regression to estimate the odds ratio (OR).

All models were adjusted for DOPPS phase, country, age, sex, Black race (due to the differences in outcomes reported in this population [28]), <90 days (versus 90–365 days) dialysis vintage, catheter use, body mass index (BMI), serum albumin, haemoglobin and 13 summary comorbid conditions: coronary artery disease, cerebrovascular disease, congestive HF, peripheral vascular disease, other cardiovascular disease, diabetes, cancer (non-skin), hypertension, lung disease, neurologic disease, psychiatric disorder, gastrointestinal bleeding and recurrent cellulitis/gangrene. Models were repeated for three secondary exposures parameterizing diuretic prescription at the facility level: facility proportion of patients prescribed a diuretic, facility mean loop diuretic dose among loop diuretic users and facility mean loop diuretic dose (including non-users as 0 dose). Models using a facility-level exposure were additionally adjusted for potential facility-level confounders [26], including facility percent catheter use, facility percent haemoglobin <10 g/dl and facility percent single-pool *Kt*/*V* <1.2.

## RESULTS

### Diuretic prescription by dialysis vintage

**Figure 1:**
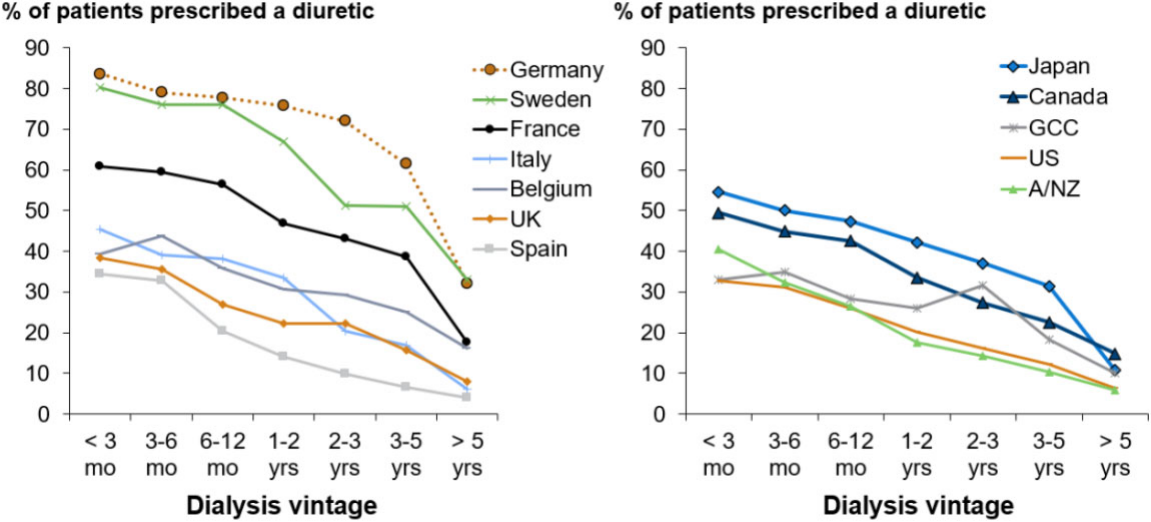
Proportion of patients prescribed a diuretic, by country and dialysis vintage. Figure split by Europe versus non-Europe for visual clarity.

The percentage of patients prescribed a diuretic at DOPPS enrolment was lower among patients who enrolled in DOPPS with a longer dialysis vintage, and this pattern was consistent across all countries. However, a wide variation in prescription prevalence at each dialysis vintage was observed across countries. The country with the highest proportion of patients treated with diuretics was Germany (almost 84% of patients before the first year of dialysis), versus Spain, where only 24% of the patients were prescribed diuretics between 6 and 12 months of dialysis vintage, which was comparable to the prescription in the USA (Fig. 1). After 5 years on dialysis, the prescription of diuretics ranged from <10% in the USA, New Zealand and Australia, Spain and Italy, to ≈30% in Germany and Sweden. Among patients who enrolled in DOPPS at or near dialysis initiation, the rate of diuretic prescription was 54%, compared with 38% for those with versus without RUV at enrolment. Among patients who enrolled in DOPPS at or near 5 years after initiating dialysis, the rate of diuretic prescription was 38%, compared with 9% for patients with versus without RUV at enrolment (Supplementary Fig. S2).

### Diuretic prescription practices in the first year of dialysis

**Figure 2:**
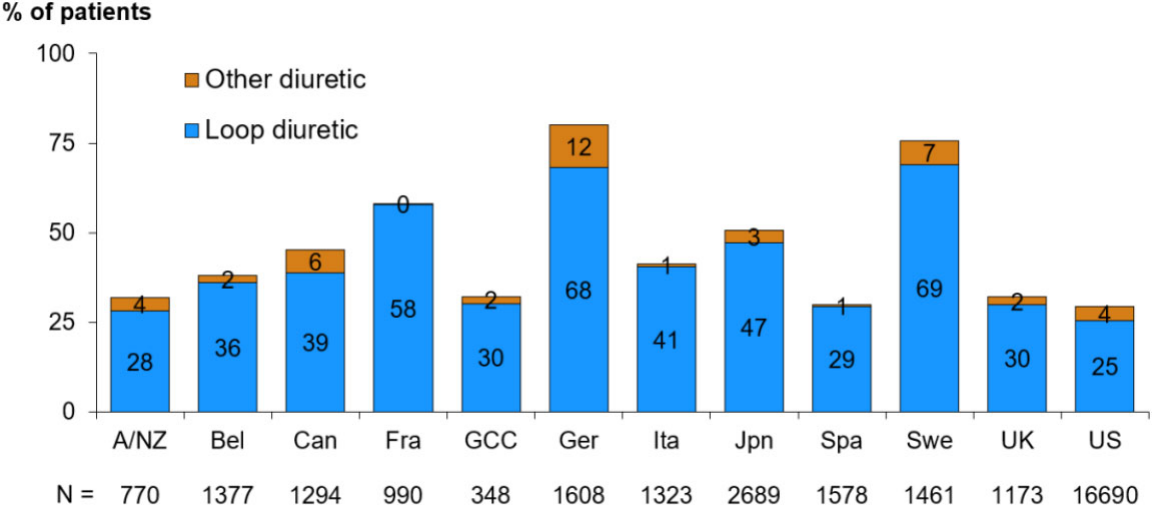
Proportion of patients prescribed a diuretic, by country and diuretic type, restricted to patients with dialysis vintage <1 year. A/NZ: Australia/New Zealand; Bel: Belgium; Can: Canada; Fra: France; GCC: Gulf Cooperation Council (Bahrain, Kuwait, Oman, Qatar, Saudi Arabia, United Arab Emirates); Ger: Germany; Ita: Italy; Jpn: Japan; Spa: Spain; Swe: Sweden; UK: United Kingdom; US: United States.

Loop diuretics were the most prescribed diuretic therapy throughout all the countries (Fig. 2) and regions (Supplementary  Fig. S3), with a prescription of other diuretics ranging from 0 to 12% of patients. There was no major change in diuretic prescriptions in the different DOPPS phases in North America and Japan, whereas the rate of prescription tended to be higher in the latest DOPPS phases in Europe (Supplementary Fig. S3). Interestingly, the countries with the overall highest proportion of diuretic prescription, namely Germany and Sweden, were also those with the narrowest range of prescriptions according to the facility {84% [interquartile range (IQR) 72–90] and 77% [IQR 68–85], respectively} (Supplementary Fig. S4).

**Figure 3:**
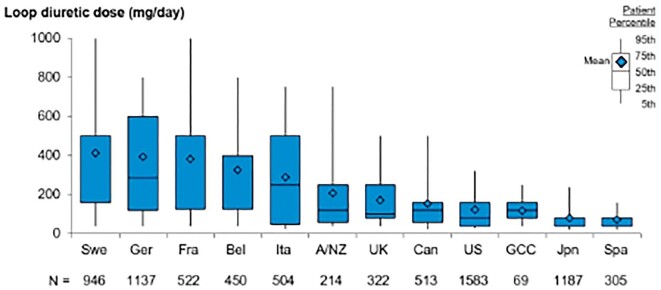
Loop diuretic dose, by country, restricted to loop diuretic users with dialysis vintage <1 year. Doses of torsemide (4:1) and bumetanide (80:1) were converted to oral furosemide-equivalent doses. A/NZ: Australia/New Zealand; Bel: Belgium; Can: Canada; Fra: France; GCC: Gulf Cooperation Council (Bahrain, Kuwait, Oman, Qatar, Saudi Arabia, United Arab Emirates); Ger: Germany; Ita: Italy; Jpn: Japan; Spa: Spain; Swe: Sweden; UK: United Kingdom; US: United States.

Not only the rate of prescription, but also the dose of loop diuretics varied widely during the first year of HD (Fig. 3). Despite wide ranges of doses, the countries with the highest rate of overall diuretic prescription were also those with the highest median daily doses prescribed, represented by Germany [286 (IQR 120–600)], Sweden [500 (IQR 160–500)] and France [500 (IQR 125–500)]. Along the same line, the Gulf Cooperation Council (GCC; Bahrain, Kuwait, Oman, Qatar, Saudi Arabia, United Arab Emirates), Spain and the USA, which had the lowest prescription rates, also had the lowest median daily doses [120 (IQR 80–160), 40 (IQR 40–80) and 80 (IQR 40–160)], respectively]. The same pattern was observed when analysing the range of mean diuretic daily dose according to the facility (Supplementary Fig. S5). An exception was Japan, which displayed one of the highest rates of diuretic prescription along with one of the lowest doses.

### Patient characteristics by diuretic prescription

Among patients who enrolled in DOPPS within the first year after initiating dialysis, patients prescribed versus not prescribed a diuretic at study enrolment were older (65.2 versus 63.9 years) and suffered more frequently from cardiovascular diseases such as coronary heart disease (35% versus 26%), HF (30% versus 24%) and hypertension (81% versus 72%) and from diabetes (57% versus 47%) (Table 1). Among patients treated with loop diuretics, a majority of patients were prescribed furosemide (79.9%) and 6.8% of patients were treated with a combination of at least two diuretics. Results were similar when comparing patients with the highest loop diuretic doses (>200 mg/day) to lower doses (60–200 mg/day and <60 mg/day) (Supplementary Table S1). Except for diabetes, the same tendency was also seen when comparing the facilities with the highest diuretic prescription rate (>60%) to the lowest (20–60% and <20%) (Supplementary Table S2) and when comparing the facilities with the highest mean diuretic dose (>200 mg/day) to the lowest mean doses (20–200 mg/day and <20 mg/day), although less conspicuous (Supplementary Table S3).

**Table 1:** Baseline patient characteristics, by diuretic use, restricted to patients with dialysis vintage <1 year.

		Diuretic use	
Characteristics	All	No	Yes
Patients, *n*	31 621	19 551	12 070
Age (years), mean (SD)	64.4 (15.0)	63.9 (15.4)	65.2 (14.3)
Male, %	59	58	61
Black race, %	13	16	10
BMI (kg/m^2^), mean (SD)	27.0 (6.6)	26.7 (6.5)	27.5 (6.6)
Vintage <90 days, %	42	39	47
Catheter use, %	49	52	44
Coronary artery disease, %	30	26	35
HF, %	26	24	30
Cerebrovascular disease, %	9	8	11
Other cardiovascular disease, %	20	17	25
Cancer (non-skin), %	10	10	10
Diabetes, %	50	47	57
Gastrointestinal bleeding, %	4	4	4
Hypertension, %	76	72	81
Lung disease, %	10	9	11
Neurologic disease, %	7	7	8
Psychiatric disorder, %	16	16	16
Recurrent cellulitis/gangrene, %	5	4	6
HIV/AIDS, %	1	1	0
Loop diuretic used			
Furosemide			79.9
Torsemide			10.4
Bumetanide			7.4
Other			2.3
Diuretic combination			6.8
Serum albumin (g/dl), mean (SD)	3.5 (0.6)	3.5 (0.6)	3.5 (0.6)
Haemoglobin (g/dl), mean (SD)	10.7 (1.6)	10.7 (1.6)	10.7 (1.5)

HIV: human immunodeficiency virus; AIDS: acquired immunodeficiency syndrome; SD: standard deviation.

### Diuretic prescription and mortality/hospitalization outcomes

The prescription of diuretics in the first year of HD was not associated with any of the analysed clinical outcomes, including all-cause mortality, all-cause hospitalization or hospitalization due to MACE and/or HF (Table 2). Only the prescription of low doses of loop diuretics (<60 mg/day) was associated with a higher risk of hospitalization due to MACE and HF [HR 1.20 (95% CI 1.05–1.37)] compared with the reference group with no diuretic treatment. Prescriptions of intermediate doses (60–200 mg/day) and high doses (>200 mg/day) were associated with higher rates of all-cause hospitalization [1.08 (95% CI 1.01–1.16) and 1.11 (95% CI 1.03–1.18), respectively], but not with mortality, after adjustment for multiple confounding factors including a history of cardiovascular diseases (Table 2) compared with the reference group. The HD facilities with the lowest diuretic prescription prevalence (<20%) tended to have the lowest rates of these adverse events (Supplementary Table S4). A sensitivity analysis was performed by restricting the analysis to patients with no RUV and found the same tendency as in the general population (Supplementary Table S5).

**Table 2:** Association between diuretic prescription and mortality/hospitalization outcomes, restricted to patients with dialysis vintage <1 year.

Variables	*n* (%)	All-cause mortality	All-cause hospitalization	Hospitalization due to MACE + HF	Hospitalization due to HF	Hospitalization due to fracture
Events, *n*		3078	8648	2629	936	421
Event rate per 100 patient-years		10.6	45.0	9.5	3.3	1.5
Patient diuretic use, HR (95% CI)						
Yes	12 070 (38.2)	1.04 (0.95–1.13)	1.03 (0.98–1.08)	1.06 (0.97–1.15)	1.06 (0.92–1.23)	0.88 (0.71–1.09)
No	19 551 (61.8)	1 (Ref.)	1 (Ref.)	1 (Ref.)	1 (Ref.)	1 (Ref.)
Patient loop diuretic dose (mg/day), HR (95% CI)						
No dose	20 234 (72.3)	1 (Ref.)	1 (Ref.)	1 (Ref.)	1 (Ref.)	1 (Ref.)
0–≤60	2084 (7.5)	1 (0.87–1.16)	1.03 (0.96–1.11)	1.2 (1.05–1.37)	1.22 (0.97–1.54)	0.94 (0.67–1.33)
60–≤200	2827 (10.1)	1.02 (0.91–1.15)	1.08 (1.01–1.16)	1.06 (0.94–1.2)	1.13 (0.93–1.37)	0.77 (0.55–1.06)
>200	2853 (10.2)	1.13 (1–1.26)	1.11 (1.03–1.18)	1.05 (0.92–1.18)	1.06 (0.85–1.32)	1.04 (0.78–1.4)

HR (95% CI) of each outcome shown for diuretic use (yes versus no) and loop diuretic dose (reference group: no dose). Doses of torsemide (4:1) and bumetanide (80:1) were converted to oral furosemide-equivalent doses. Cox models stratified by DOPPS phase and country and adjusted for age, sex, Black race, <90 days dialysis vintage, catheter use, BMI, serum albumin, haemoglobin and 13 comorbidities.

### Diuretic prescription and MBD outcomes

Based on previous evidence of a higher risk of secondary hyperparathyroidism in patients treated with loop diuretics [21, 22], we analysed the association between loop diuretic prescription and dose and MBD outcomes. Unexpectedly, diuretic prescription was associated with lower circulating PTH [−11.6 pg/ml (95% CI −20.6 to −2.5)] and total ALP levels [−2.3 IU/l (95% CI −4.8 to −0.3)] in multivariable analyses, although with no associations according to the diuretic dose (Table 3). In the facility-based approach, the same results were seen for total ALP, with lower levels in facilities with the highest rate of diuretic prescription [>60%; mean difference −13.3 IU/l (95% CI −20.1 to −6.5)] and with the highest mean loop diuretic dose [>200 mg/day; mean difference −8.4 IU/l (95% CI −18.1–1.4)] (Supplementary Table S6). A higher mean serum calcium level was observed in the facilities with the lowest rate of diuretic prescription [<20%; mean difference 0.05 mg/dl (95% CI 0.02–0.09)] and those with the lowest mean diuretic dose [<20 mg/day; mean difference 0.05 mg/dl (95% CI 0.01–0.08)] (Supplementary Table S6). Moreover, no increased risk of hospitalization due to fracture was observed across loop diuretic dose categories (Table 2).

**Table 3:** Association between diuretic prescription and MBD outcomes, restricted to patients with dialysis vintage <1 year.

Variables	*n* (%)	Serum calcium (mg/dl)	Serum phosphorus (mg/dl)	PTH (pg/ml)	Total ALP (IU/l)
Patients, *n*		29 350	29 515	24 617	24 533
Mean (SD)		8.82 (0.8)	5.08 (1.64)	315.9 (330.8)	134.2 (204.6)
Patient diuretic use, mean (95% CI)					
Yes	38.2	−0.01 (−0.03–0.01)	0.03 (−0.01–0.07)	−11.6 (−20.6 to −2.5)	−2.3 (−4.8–0.3)
No	61.8	0 (Ref.)	0 (Ref.)	0 (Ref.)	0 (Ref.)
Patient loop diuretic dose (mg/day), mean (95% CI)					
No dose	72.3	0 (Ref.)	0 (Ref.)	0 (Ref.)	0 (Ref.)
0–≤60	7.5	−0.02 (−0.05–0.02)	0.02 (−0.06–0.1)	−5.8 (−24.1–12.4)	−2 (−7.1–3)
60–≤200	10.1	−0.01 (−0.04–0.02)	0.05 (−0.02–0.12)	−10.7 (−26.5–5.2)	−2.2 (−6.5–2.2)
>200	10.2	−0.02 (−0.05–0.02)	0.1 (0.02–0.18)	0.5 (−17.8–18.7)	1.1 (−4.2–6.4)

Mean difference (95% CI) of each outcome shown for diuretic use (yes versus no) and loop diuretic dose (reference group: no dose). Doses of torsemide (4:1) and bumetanide (80:1) were converted to oral furosemide-equivalent doses. Linear mixed models adjusted for DOPPS phase, country, age, sex, Black race, <90 days dialysis vintage, catheter use, BMI, serum albumin, haemoglobin and 13 comorbidities.

### Diuretic prescription and extracellular volume outcomes

The prescription of diuretics was associated with a slightly higher pre-dialysis SBP [mean difference 1.0 mmHg (95% CI 0.4–1.5)] compared with patients with no prescription (Table 4). However, no dose–response pattern was observed, and minimal association was found between diuretic use/dose and DBP. A lower IDWG was associated with low loop diuretic doses [<60 mg/day; mean difference −0.30 kg (95% CI −0.47 to −0.13)]. There was also an association between the proportion of patients with an RUV >200 ml/day and the diuretic prescription [OR 1.51 (95% CI 1.41–1.61)], as well as with all the categories of diuretic dose prescribed (Table 4). This was confirmed in the facility-based approach when analysing the facility proportion of patients prescribed a loop diuretic [>60% versus 20–60% use; OR 1.43 (95% CI 1.15–1.78)] or the facility mean dose [mean daily dose >200 mg/day; OR 1.73 (95% CI 1.31–2.27)] (Supplementary Table S7).

**Table 4:** Association between diuretic prescription and extracellular volume outcomes, restricted to patients with dialysis vintage <1 year.

Variables	*n* (%)	Pre-dialysis SBP (mmHg)	Pre-dialysis DBP (mmHg)	IDWG (kg)	RUV (as >200 ml/24 h)
Patients, *n*		29 934	29 923	27 538	31 621
Mean (SD)		143.9 (22.55)	75.14 (13.62)	2.49 (3.31)	
Events, *n*					8382
Prevalence of outcome, %					26.5
Patient diuretic use, mean (95% CI)					
Yes	38.2	1 (0.4–1.5)	−0.2 (−0.5–0.1)	−0.09 (−0.18 to −0.01)	1.51 (1.41–1.61)
No	61.8	0 (Ref.)	0 (Ref.)	0 (Ref.)	1 (Ref.)
Patient loop diuretic dose (mg/day), mean (95% CI)					
No dose	72.3	0 (Ref.)	0 (Ref.)	0 (Ref.)	1 (Ref.)
0–≤60	7.5	1.2 (0.1–2.2)	−0.3 (−0.9–0.3)	−0.3 (−0.47 to −0.13)	1.5 (1.34–1.68)
60–≤200	10.1	0.2 (−0.8–1.1)	−0.7 (−1.2, -0.2)	−0.11 (−0.26–0.04)	1.59 (1.43–1.76)
>200	10.2	0.6 (−0.5–1.7)	−0.4 (−1–0.2)	−0.01 (−0.19–0.16)	1.59 (1.42–1.78)

Mean difference (95% CI) or odds ratio (95% CI) of each outcome shown for diuretic use (yes versus no) and loop diuretic dose (reference group: no dose). Doses of torsemide (4:1) and bumetanide (80:1) were converted to oral furosemide-equivalent doses. Linear mixed models (for SBP, DBP and IDWG) and logistic regression models (for RUV) adjusted for DOPPS phase, country, age, sex, Black race, <90 days dialysis vintage, catheter use, BMI, serum albumin, haemoglobin and 13 comorbidities.

## DISCUSSION

Our study showed that the rate of prescription and daily dose of loop diuretics varied widely according to country and region, with very high rates and doses (up to 1500 mg/day) in several European countries, in particular Germany and Sweden. Despite an association with diuresis conservation, the prescription of diuretics in general, and the prescription of high doses in particular, was not associated with improved outcomes, including all-cause mortality or MACE, but with a higher risk of all-cause hospitalization with intermediate (60–200 mg/day) and high doses (>200 mg/day). However, they were not associated with adverse MBD events, including hospitalization for fractures or higher serum PTH levels.

As demonstrated by the high variation according to country and region, the prescription and dose of loop diuretics in CKD patients initiating HD seems mainly determined by an empirical approach. A high variability in prescription patterns in the USA was also recently reported by Flythe and Assimon [29]. This might be at least partly related to the lack of solid evidence supporting their use, with conflicting results in previous literature on their association with outcomes in patients undergoing HD. Of note, no substantial difference in loop diuretic use was observed in the different DOPPS phases, demonstrating the absence of a global change in prescription in the past 25 years. Regarding the dose, the pharmacodynamics of loop diuretics support the use of higher doses when the GFR decreases and explains the renal resistance at conventional doses in most patients with advanced CKD [16, 30]. Diuretic resistance also occurs when the ceiling dose is reached [31], potentially explaining the lack of association between very high doses (>500 mg/day) and beneficial outcomes in our study. Our observation of a progressive decrease in diuretic prescription with dialysis vintage is consistent with previous literature [1], including a study in the earlier phases of DOPPS by Bragg-Gresham *et al*. [19].

In contrast to the encouraging results for the use of diuretics in HD patients in the studies by Bragg-Gresham *et al*. and Sibbel *et al*. [19, 20], our study failed to demonstrate a beneficial effect of diuretic prescription, even at relatively high doses, on adverse outcomes such as MACE and mortality. The reverse was observed, with a higher risk of all-cause hospitalization in patients with a diuretic prescription. The analysis was conducted after adjustment for multiple potential confounding factors, including a history of cardiovascular diseases, that might lead to an increase in diuretic prescription. Nevertheless, residual confounding factors yet to be identified might explain this result. Based on the characteristics of patients treated with diuretic prescription in our study, one can hypothesize that the negative results observed could be explained by the fact that these patients had a generally more severe condition, namely they were 1.3 years older, had 6% more HF and had 7% more ‘other’ cardiovascular diseases. Previous interventional studies tend to support the use of loop diuretics in the HD population, even at high doses, even though the population sample has been relatively small [32, 33]. Our study also addressed the potential harmfulness of loop diuretics on MBD outcomes. Patients prescribed diuretics did not have higher serum PTH levels or hospital admission rates due to fractures compared with those without diuretic prescription, in contrast to other reports [21, 22]. This discrepancy might be explained by the low urinary calcium excretion in CKD stage 5D even when treated with loop diuretics [34].

Despite the adverse signal on the all-cause hospitalization outcome, patients treated with loop diuretics, and even in those with the highest doses, had a greater chance of preserved RUV; this was confirmed in the facility-level multivariable analysis, suggesting that diuretic prescription may help conserve RUV. However, a reverse causality effect cannot be ruled out, as diuretics might be maintained only in patients with RUV. Moreover, higher doses of diuretics were associated with higher IDWG, suggesting that the latter might be the primary driver of the prescription of higher doses of diuretics. In the open-label study by Flythe *et al*. [32], only one-third of the 36 patients treated with variable doses of oral furosemide (up to 320 mg/day) increased their RUV, achieving a 24-h urine output >200 ml (from 227 to 322 ml/24 h and GFR from 1.3 to 1.6 ml/min), with no difference in organic solutes clearance from baseline to 5 weeks after treatment initiation [35], while Siga and Alcuaz [33] showed that 33 of 34 patients in their randomized crossover trial testing 250 and 500 mg/day furosemide maintained their RUV after 1 year of treatment. In our study, no effect of high doses of loop diuretics was observed on IDWG.

Our study has some limitations. First, due to the observational design of the study and the absence of compliance control, causality cannot be shown. Second, the classification of residual urinary volume was not based on 24-h urine collections, thus increasing the likelihood of misclassification bias. Third, diuretic prescription at or soon after dialysis initiation might reflect physician practice patterns prior to initiating dialysis and not those of the HD facility; data on diuretic prescription prior to DOPPS enrolment was unfortunately not available in this database. Fourth, while diuretic prescription was captured periodically during follow-up (every 4 months), exact start/stop dates were unknown, so we chose to utilize only the baseline prescription for purposes of this analysis. Fifth, extracellular volume assessments were imperfect, as they relied on measured BP and IDWG. Sixth, notwithstanding multiple adjustments for confounding factors such as comorbidities and the use of a facility-based approach to confirm our findings [36], an indication bias likely affected the results, as patients treated with diuretics were older and more often had a diagnosis of diabetes and cardiovascular disease. In the clinical setting of HD, where diuretic therapy is routinely prescribed, the issue of indication bias is of major importance, similar to the situation of patients with HF. Stronger evidence with well-designed clinical trials is necessary to draw conclusions on the benefit of diuretics (and higher doses) in these patients [37]. Seventh, missing values for the variables of interest might also account for a potential additional bias. Finally, no data were available regarding ototoxicity, diabetes onset and skin photoreaction, which might be a concern when patients are treated with high doses of loop diuretics. Epidemiological data suggest a causal role of loop diuretics in the risk of long-term hearing loss [38, 39]. However, previous reports of experimental animal models suggest that hearing loss results from acute doses and is reversible [40].

In conclusion, neither the prescription of loop diuretics nor the prescription of high doses of loop diuretics were associated with any of the beneficial outcomes that were measured in our study. On the contrary, higher doses were associated with an increased risk of all-cause hospitalizations. Given the additional risk of undesirable side effects of high-dose prescriptions of loop diuretics, and in the light of the high variability in dosing and prescription across the countries and regions included in the DOPPS, our results might help inform future clinical trials to harmonize the prescription of loop diuretics in HD patients.

## Supplementary Material

sfae141_Supplemental_File

## Data Availability

The data that support the findings of this study are available from the corresponding author upon reasonable request.
