# Supplementary material for: Diuretic prescriptions in the first year of haemodialysis: international practice patterns and associations with outcomes
Source: Clin Kidney J. 2024 Jun 14;17(7):sfae141. doi: 10.1093/ckj/sfae141 (PMC11233991; doi:10.1093/ckj/sfae141)
Supplement: sfae141_Supplemental_File [file sfae141_supplemental_file.docx]

**Diuretic prescriptions in the first year of hemodialysis: International practice patterns and associations with outcomes**

## Supplementary Material

## Table of contents

**Suppl Figure S1**. Consort diagram of the study population.

**Suppl Figure S2**. Proportion of patients prescribed a diuretic, by residual urine volume and dialysis vintage. RUV=Residual urine volume, defined as urine output >200 mL/day.

**Suppl Figure S3.** Proportion of patients prescribed a diuretic, by DOPPS phase a region, restricted to patients with dialysis vintage <1 year

**Suppl Figure S4.** Facility proportion of patients prescribed a diuretic, by country, restricted to patients with dialysis vintage <1 year

**Suppl Figure S5.** Facility mean loop diuretic dose, by country, restricted to loop diuretic users with dialysis vintage <1 year

**Suppl Table S1**. Baseline patient characteristics, by loop diuretic dose, restricted to loop diuretic users with dialysis vintage <1 year

**Suppl Table S2.** Baseline patient characteristics, by facility proportion of patients prescribed a diuretic, restricted to patients with dialysis vintage <1 year

**Suppl Table S3.** Baseline patient characteristics, by facility mean loop diuretic dose, restricted to loop diuretic users with dialysis vintage <1 year

**Suppl Table S4**. Association between facility-level diuretic prescription and mortality/hospitalization outcomes, restricted to patients with dialysis vintage <1 year

**Suppl Table S5.** Association between diuretic prescription and mortality/hospitalization outcomes, restricted to patients with dialysis vintage <1 year and <200 mL/day residual urine volume

**Suppl Table S6.** Association between facility-level diuretic prescription and mineral and bone disorder outcomes, restricted to patients with dialysis vintage <1 year

**Suppl Table S7.** Association between facility-level diuretic prescription and extracellular volume outcomes, restricted to patients with dialysis vintage <1 year

## Suppl Figure S1

**Suppl Figure S1. Consort diagram of the study population**


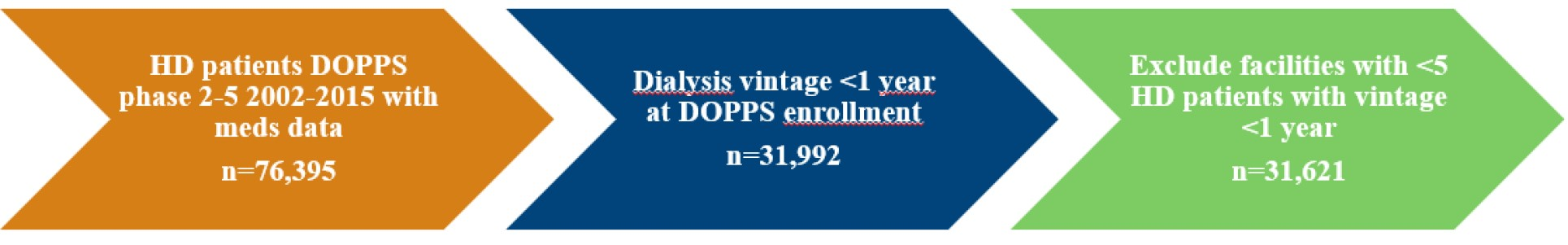


## Suppl Figure S2

**Suppl Figure S2. Proportion of patients prescribed a diuretic, by residual urine volume and dialysis vintage**


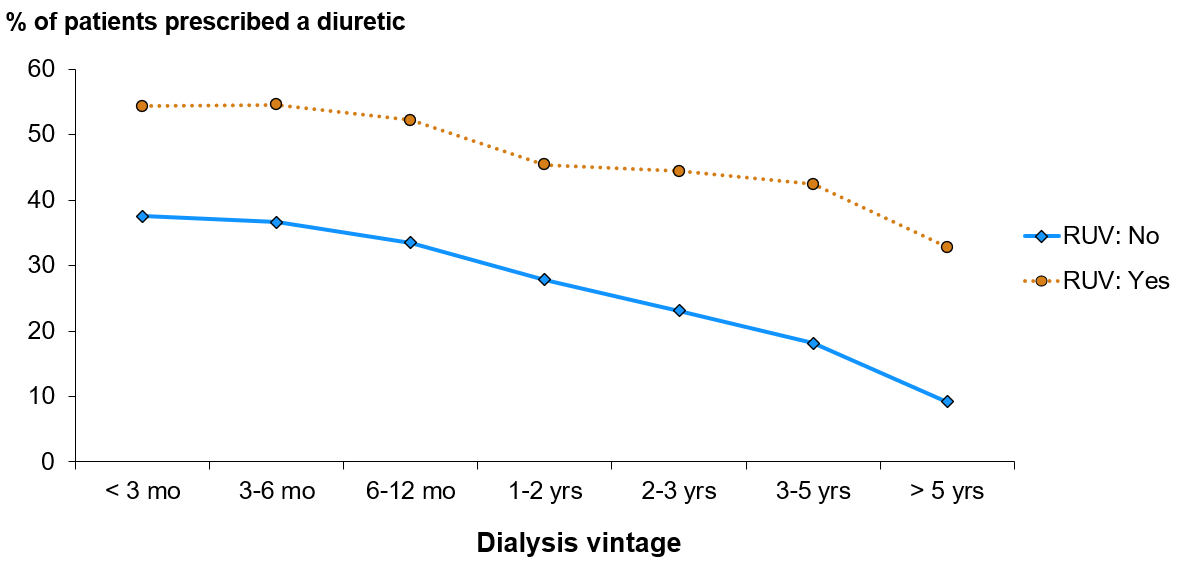


## Suppl Figure S3

**Suppl Figure S3. Proportion of patients prescribed a diuretic, by diuretic type, DOPPS phase, and region, restricted to patients with dialysis vintage <1 year**


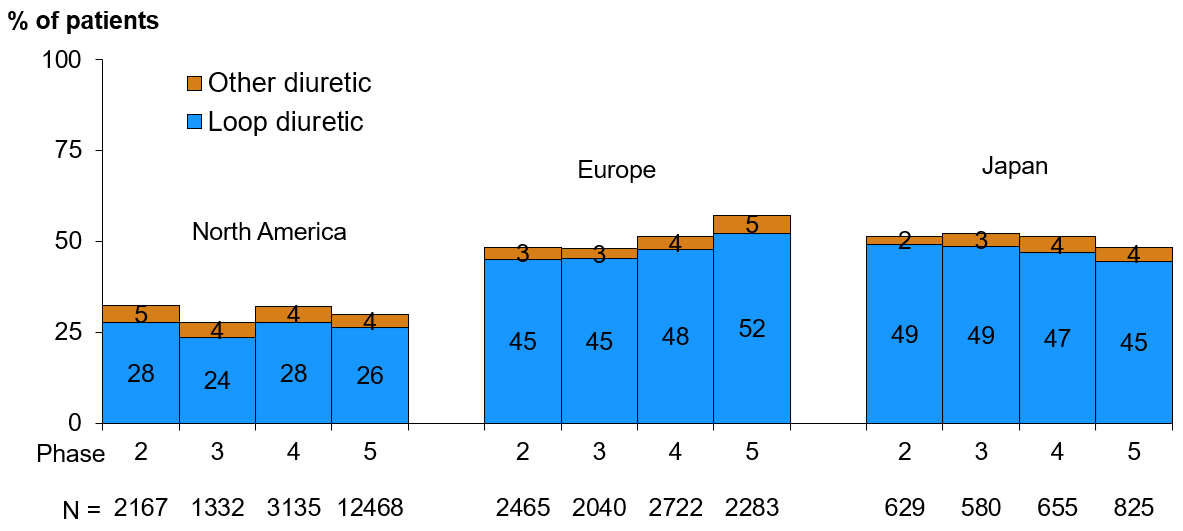


## Suppl Figure S4

**Suppl Figure S4. Facility proportion of patients prescribed a diuretic, by country, restricted to patients with dialysis vintage <1 year**


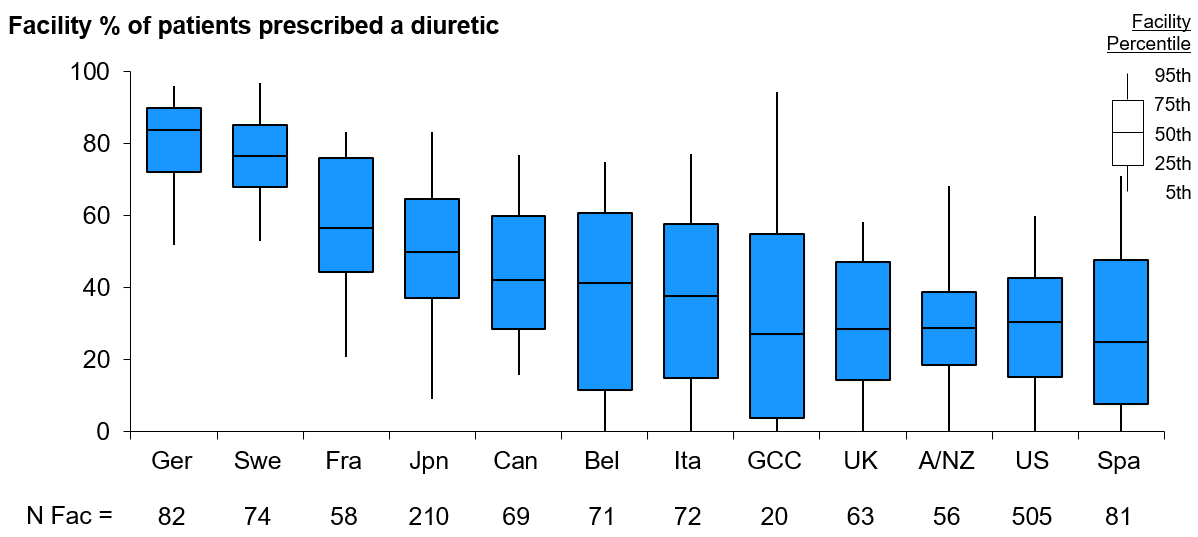


## Suppl Figure S5

**Suppl Figure S5. Facility mean loop diuretic dose, by country, restricted to loop diuretic users with dialysis vintage <1 year**


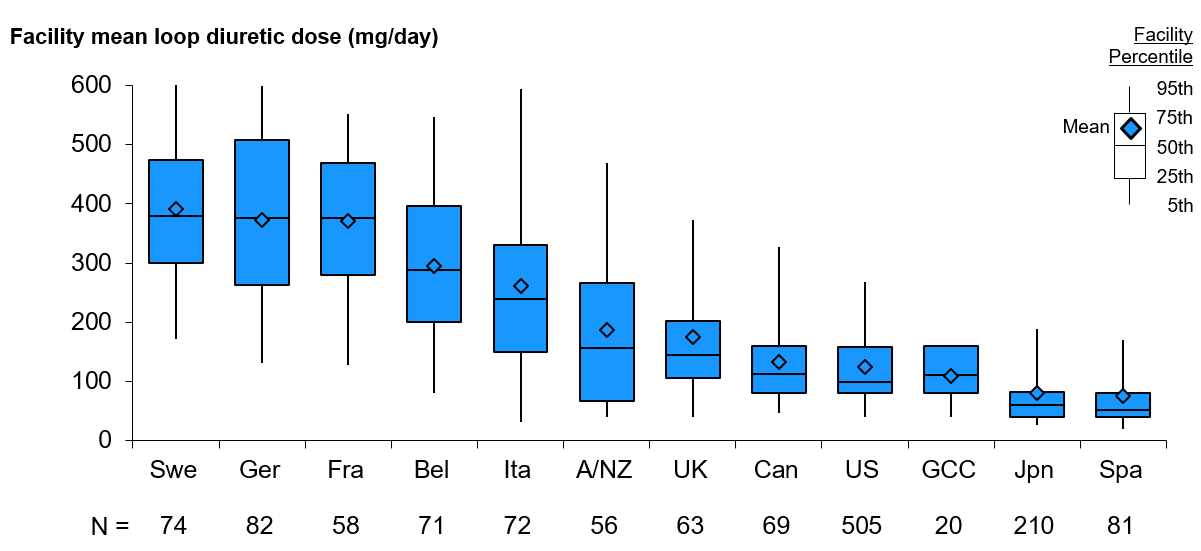


## Suppl Table S1

**Suppl Table S1. Baseline patient characteristics, by loop diuretic dose, restricted to loop diuretic users with dialysis vintage <1 year**

|  | Patient loop diuretic dose | | | |
| --- | --- | --- | --- | --- |
| Characteristics | no dose | 0<-60mg/day | 60<-200mg/day | >200mg/day |
| Number of patients | 20,234 | 2,084 | 2,827 | 2,853 |
| Age | 63.9 (15.4) | 65.1 (14.0) | 64.8 (14.4) | 65.6 (14.0) |
| Male Sex | 58% | 62% | 61% | 64% |
| Black race | 16% | 5% | 9% | 3% |
| Vintage less than 90 days | 39% | 53% | 52% | 50% |
| BMI, kg/m2 | 26.7 (6.6) | 25.4 (5.9) | 27.2 (6.6) | 27.2 (6.1) |
| Serum Albumin, g/dL | 3.5 (0.6) | 3.6 (0.5) | 3.6 (0.5) | 3.5 (0.6) |
| Hemoglobin, g/dL | 10.7 (1.6) | 10.7 (1.6) | 10.7 (1.6) | 10.9 (1.6) |
| Catheter use | 52% | 29% | 41% | 42% |
| Coronary Artery Disease | 27% | 35% | 41% | 48% |
| Heart Failure | 24% | 25% | 34% | 37% |
| Cerebrovascular Disease | 8% | 13% | 14% | 16% |
| Other Cardiovascular Disease | 17% | 25% | 28% | 37% |
| Cancer (non-skin) | 10% | 12% | 12% | 15% |
| Diabetes | 47% | 49% | 58% | 53% |
| Gastrointestinal Bleeding | 4% | 4% | 4% | 6% |
| Hypertension | 72% | 87% | 86% | 89% |
| Lung Disease | 9% | 10% | 13% | 14% |
| Neurologic Disease | 7% | 10% | 9% | 10% |
| Psychiatric Disorder | 16% | 13% | 16% | 15% |
| Recurrent Cellulitis, Gangrene | 4% | 5% | 7% | 11% |
| HIV / AIDS | 1% | 0% | 0% | 0% |

Mean (standard deviation) or prevalence (%) shown.

## Suppl Table S2

**Suppl Table S2. Baseline patient characteristics, by facility proportion of patients prescribed a diuretic, restricted to patients with dialysis vintage <1 year**

|  | Facility % diuretic use | | |
| --- | --- | --- | --- |
| Characteristics | <20% | 20%-<60% | ≥60% |
| Number of patients | 7,612 | 17,951 | 6,058 |
| Age | 64.1 (15.4) | 64.1 (15.0) | 65.4 (14.5) |
| Male Sex | 57% | 59% | 63% |
| Black race | 17% | 16% | 2% |
| Vintage less than 90 days | 36% | 43% | 47% |
| BMI, kg/m2 | 26.8 (6.6) | 27.4 (6.8) | 25.9 (5.7) |
| Serum Albumin, g/dL | 3.5 (0.6) | 3.6 (0.5) | 3.5 (0.6) |
| Hemoglobin, g/dL | 10.7 (1.6) | 10.7 (1.5) | 10.8 (1.6) |
| Catheter use | 52% | 52% | 37% |
| Coronary Artery Disease | 26% | 28% | 39% |
| Heart Failure | 25% | 26% | 28% |
| Cerebrovascular Disease | 8% | 8% | 14% |
| Other Cardiovascular Disease | 17% | 18% | 30% |
| Cancer (non-skin) | 8% | 9% | 15% |
| Diabetes | 48% | 53% | 45% |
| Gastrointestinal Bleeding | 4% | 3% | 5% |
| Hypertension | 70% | 75% | 85% |
| Lung Disease | 9% | 10% | 12% |
| Neurologic Disease | 6% | 7% | 10% |
| Psychiatric Disorder | 14% | 18% | 13% |
| Recurrent Cellulitis, Gangrene | 4% | 5% | 7% |
| HIV / AIDS | 1% | 0% | 0% |

Mean (standard deviation) or prevalence (%) shown.

## Suppl Table S3

**Suppl Table S3. Baseline patient characteristics, by facility mean loop diuretic dose, restricted to loop diuretic users with dialysis vintage <1 year**

|  | Facility mean loop diuretic dose, including non-users as 0 dose | | |
| --- | --- | --- | --- |
| Characteristics | <20mg/day | 20-<200mg/day | ≥200mg/day |
| Number of patients | 16,999 | 11,153 | 3,469 |
| Age | 64.3 (15.2) | 64.1 (14.8) | 65.6 (14.7) |
| Male Sex | 58% | 60% | 63% |
| Black race | 20% | 8% | 2% |
| Vintage less than 90 days | 38% | 47% | 48% |
| BMI, kg/m2 | 27.5 (6.9) | 26.3 (6.4) | 26.3 (5.4) |
| Serum Albumin, g/dL | 3.5 (0.5) | 3.6 (0.6) | 3.5 (0.6) |
| Hemoglobin, g/dL | 10.6 (1.5) | 10.7 (1.6) | 10.9 (1.6) |
| Catheter use | 56% | 42% | 39% |
| Coronary Artery Disease | 22% | 37% | 43% |
| Heart Failure | 24% | 29% | 29% |
| Cerebrovascular Disease | 5% | 13% | 16% |
| Other Cardiovascular Disease | 13% | 26% | 35% |
| Cancer (non-skin) | 6% | 14% | 18% |
| Diabetes | 53% | 49% | 41% |
| Gastrointestinal Bleeding | 3% | 5% | 6% |
| Hypertension | 69% | 82% | 87% |
| Lung Disease | 8% | 12% | 13% |
| Neurologic Disease | 5% | 9% | 11% |
| Psychiatric Disorder | 16% | 17% | 14% |
| Recurrent Cellulitis, Gangrene | 3% | 7% | 8% |
| HIV / AIDS | 1% | 0% | 1% |

## Suppl Table S4

**Suppl Table S4. Association between facility-level diuretic prescription and mortality/hospitalization outcomes, restricted to patients with dialysis vintage <1 year**

|  | **N(%)** | **All-cause mortality** | **All-cause hospitalization** | **Hospitalization due to MACE+HF** | **Hospitalization due to HF** | **Hospitalization due to fracture** |
| --- | --- | --- | --- | --- | --- | --- |
| **No. of Events** |  | 3,078 | 8,648 | 2,629 | 936 | 421 |
| **Event rate per 100 pat-yrs** |  | 10.6 | 45.0 | 9.5 | 3.3 | 1.5 |
| **Facility proportion of patients prescribed a diuretic** | | | | | | |
| <20% | 7,612 (24.1%) | 0.89  (0.79, 1.01) | 0.88  (0.8, 0.97) | 0.85  (0.74, 0.97) | 0.77  (0.62, 0.96) | 0.89  (0.67, 1.19) |
| 20%-<60% | 17,951 (56.7%) | 1 (Ref.) | 1 (Ref.) | 1 (Ref.) | 1 (Ref.) | 1 (Ref.) |
| >=60% | 6,058 (19.2%) | 1.01  (0.89, 1.13) | 0.97  (0.89, 1.06) | 0.96  (0.85, 1.1) | 0.95  (0.76, 1.19) | 0.9  (0.66, 1.21) |
| **Facility mean loop diuretic dose, including non-users as 0 dose** | | | | | | |
| <20mg/day | 16,999 (53.8%) | 0.97  (0.86, 1.08) | 0.91  (0.83, 0.98) | 0.88  (0.78, 0.99) | 0.91  (0.74, 1.1) | 0.74  (0.57, 0.95) |
| 20-<200mg/day | 11,153 (35.3%) | 1 (Ref.) | 1 (Ref.) | 1 (Ref.) | 1 (Ref.) | 1 (Ref.) |
| >=200mg/day | 3,469 (11%) | 0.95  (0.83, 1.08) | 1.08  (0.97, 1.21) | 0.9  (0.78, 1.03) | 1.01  (0.76, 1.34) | 0.93  (0.67, 1.3) |
| **Facility mean loop diuretic dose, among loop diuretic users** | | | | | | |
| <80mg/day | 13,283 (42.0%) | 0.94  (0.83, 1.06) | 0.93  (0.85, 1.01) | 0.99  (0.87, 1.12) | 0.92  (0.75, 1.14) | 0.89  (0.68, 1.18) |
| 80-<400mg/day | 15,892 (50.3%) | 1 (Ref.) | 1 (Ref.) | 1 (Ref.) | 1 (Ref.) | 1 (Ref.) |
| >=400mg/day | 2,446 (7.7%) | 0.99  (0.87, 1.13) | 1.02  (0.92, 1.14) | 1.07  (0.93, 1.23) | 1.17  (0.93, 1.47) | 0.98  (0.73, 1.33) |

Hazard ratio (95% CI) of each outcome shown for facility % diuretic use (Reference: 20-60%), facility mean loop diuretic dose including non-users as 0 dose (Reference: 20-200 mg/day), and facility mean loop diuretic dose among loop diuretic users. Doses of torsemide (4:1) and bumetanide (80:1) were converted to oral furosemide-equivalent dose. Cox models stratified by DOPPS phase and country, and adjusted for age, sex, Black race, <90 days dialysis vintage, catheter use, BMI, serum albumin, hemoglobin, and 13 comorbidities.

## Suppl Table S5

**Suppl Table S5. Association between diuretic prescription and mortality/hospitalization outcomes, restricted to patients with dialysis vintage <1 year and <200 mL/day residual urine volume**

|  | **N(%)** | **All-cause mortality** | **All-cause hospitalization** | **Hospitalization due to MACE+HF** | **Hospitalization due to HF** | **Hospitalization due to fracture** |
| --- | --- | --- | --- | --- | --- | --- |
| **No. of Events** |  | 1765 | 4592 | 1424 | 518 | 237 |
| **Event rate per 100 patient-year** |  | 8.5 | 32.8 | 7.1 | 2.5 | 1.2 |
| **Patient diuretic use** | | | | | | |
| Yes | 7624 (32.8%) | 1.09  (0.97, 1.22) | 1.02  (0.96, 1.1) | 1.07  (0.96, 1.2) | 1.13  (0.94, 1.36) | 0.78  (0.6, 1.03) |
| No | 15615 (67.2%) | 1 (Ref.) | 1 (Ref.) | 1 (Ref.) | 1 (Ref.) | 1 (Ref.) |
| **Patient loop diuretic dose** | | | | | | |
| No dose | 16177 (81.1%) | 1 (Ref.) | 1 (Ref.) | 1 (Ref.) | 1 (Ref.) | 1 (Ref.) |
| 0<-60mg/day | 1128 (5.7%) | 1.09  (0.9, 1.33) | 1.13  (1.02, 1.26) | 1.19  (0.98, 1.44) | 1.12  (0.82, 1.55) | 1.1  (0.7, 1.72) |
| 60<-200mg/day | 1552 (7.8%) | 0.99  (0.82, 1.19) | 1.05  (0.95, 1.16) | 1.02  (0.85, 1.22) | 1.26  (0.97, 1.63) | 0.7  (0.46, 1.06) |
| >200mg/day | 1103 (5.5%) | 1.25  (1.06, 1.47) | 1.1  (0.99, 1.22) | 1.27  (1.08, 1.49) | 1.34  (1, 1.8) | 0.83  (0.55, 1.23) |

Hazard ratio (95% CI) of each outcome shown for diuretic use (Yes vs. No) and loop diuretic dose (Reference group: No dose). Doses of torsemide (4:1) and bumetanide (80:1) were converted to oral furosemide-equivalent dose. Cox models stratified by DOPPS phase and country, and adjusted for age, sex, Black race, <90 days dialysis vintage, catheter use, BMI, serum albumin, hemoglobin, and 13 comorbidities. MACE: major adverse cardiovascular event; HF: heart failure

## Suppl Table S6

**Suppl Table S6. Association between facility-level diuretic prescription and mineral and bone disorder outcomes, restricted to patients with dialysis vintage <1 year**

|  | **N(%)** | **Serum calcium**  **(mg/dL)** | **Serum phosphorus**  **(mg/dL)** | **PTH**  **(pg/ml)** | **Total alkaline phosphatase**  **(IU/L)** |
| --- | --- | --- | --- | --- | --- |
| **No. of Patients** |  | 29,350 | 29,515 | 24,617 | 24,533 |
| **mean (SD)** |  | 8.82 (0.8) | 5.08 (1.64) | 315.9 (330.8) | 134.2 (204.6) |
| **Facility proportion of patients prescribed a diuretic** | | | | | |
| <20% | 24.1% | 0.05  (0.02, 0.09) | -0.03  (-0.1, 0.04) | -2.9  (-18, 12.3) | 3.9  (-1.7, 9.4) |
| 20%-<60% | 56.7% | 0 (Ref.) | 0 (Ref.) | 0 (Ref.) | 0 (Ref.) |
| >=60% | 19.2% | 0  (-0.04, 0.05) | -0.09  (-0.17, -0.01) | -9.6  (-28.5, 9.3) | -13.3  (-20.1, -6.5) |
| **Facility mean loop diuretic dose, including non-users as 0 dose** | | | | | |
| <20mg/day | 53.8% | 0.05  (0.01, 0.08) | -0.04  (-0.1, 0.03) | -4.4  (-18.8, 10) | 4.8  (-0.3, 10) |
| 20-<200mg/day | 35.3% | 0 (Ref.) | 0 (Ref.) | 0 (Ref.) | 0 (Ref.) |
| >=200mg/day | 11% | 0.03  (-0.03, 0.1) | -0.01  (-0.12, 0.11) | -2.1  (-28.3, 24.1) | -8.4  (-18.1, 1.4) |
| **Facility mean loop diuretic dose, among loop diuretic users** | | | | | |
| <80mg/day | 42.0% | 0.03  (-0.01, 0.06) | -0.1  (-0.16, -0.04) | -11.6  (-25.5, 2.2) | 1.5  (-3.7, 6.6) |
| 80-<400mg/day | 50.3% | 0 (Ref.) | 0 (Ref.) | 0 (Ref.) | 0 (Ref.) |
| >=400mg/day | 7.7% | 0.02  (-0.04, 0.08) | -0.06  (-0.17, 0.04) | -1.9  (-26.3, 22.4) | -0.6  (-9.8, 8.6) |

Mean difference (95% CI) of each outcome shown for facility % diuretic use (Reference: 20-60%), facility mean loop diuretic dose including non-users as 0 dose (Reference: 20-200 mg/day), and facility mean loop diuretic dose among loop diuretic users. Doses of torsemide (4:1) and bumetanide (80:1) were converted to oral furosemide-equivalent dose. Linear mixed models adjusted for DOPPS phase, country, age, sex, Black race, <90 days dialysis vintage, catheter use, BMI, serum albumin, hemoglobin, and 13 comorbidities.

## Suppl Table S7

**Suppl Table S7. Association between facility-level diuretic prescription and extracellular volume outcomes, restricted to patients with dialysis vintage <1 year**

|  | **N(%)** | **Pre-dialysis systolic blood pressure (SBP in mmHg)** | **Pre-dialysis diastolic blood pressure (DBP in mmHg)** | **Interdialytic weight gain (IDWG in kg)** | **Residual urine volume (RUV as > 200 ml/24 h)** |
| --- | --- | --- | --- | --- | --- |
| **No. of Patients** |  | 29,934 | 29,923 | 27,538 | 31,621 |
| **mean (SD)** |  | 143.9 (22.55) | 75.14 (13.62) | 2.49 (3.31) |  |
| **No. of Events** |  |  |  |  | 8382 |
| **% prevalence of outcome** |  |  |  |  | 26.5% |
| **Facility proportion of patients prescribed a diuretic** | | | | | |
| <20% | 24.1% | -1  (-2, -0.1) | -0.6  (-1.2, -0.1) | 0.15  (0.03, 0.28) | 0.6  (0.48, 0.74) |
| 20%-<60% | 56.7% | 0 (Ref.) | 0 (Ref.) | 0 (Ref.) | 1 (Ref.) |
| >=60% | 19.2% | -0.1  (-1.3, 1) | -0.8  (-1.5, -0.2) | -0.23  (-0.39, -0.07) | 1.43  (1.15, 1.78) |
| **Facility mean loop diuretic dose, including non-users as 0 dose** | | | | | |
| <20mg/day | 53.8% | 0  (-0.9, 0.9) | 0.3  (-0.3, 0.8) | 0.08  (-0.04, 0.2) | 0.62  (0.52, 0.74) |
| 20-<200mg/day | 35.3% | 0 (Ref.) | 0 (Ref.) | 0 (Ref.) | 1 (Ref.) |
| >=200mg/day | 11% | -0.1  (-1.7, 1.5) | -0.1  (-1, 0.8) | -0.25  (-0.48, -0.02) | 1.73  (1.31, 2.27) |
| **Facility mean loop diuretic dose, among loop diuretic users** | | | | | |
| <80mg/day | 42.0% | 0  (-0.8, 0.9) | 0.1  (-0.4, 0.6) | 0.02  (-0.1, 0.13) | 0.81  (0.67, 0.98) |
| 80-<400mg/day | 50.3% | 0 (Ref.) | 0 (Ref.) | 0 (Ref.) | 1 (Ref.) |
| >=400mg/day | 7.7% | 0.1  (-1.5, 1.6) | -0.1  (-1, 0.8) | 0.08  (-0.14, 0.29) | 1.13  (0.86, 1.47) |

Mean difference (95% CI) or odds ratio (95% CI) of each outcome shown for facility % diuretic use (Reference: 20-60%), facility mean loop diuretic dose including non-users as 0 dose (Reference: 20-200 mg/day), and facility mean loop diuretic dose among loop diuretic users. Doses of torsemide (4:1) and bumetanide (80:1) were converted to oral furosemide-equivalent dose. Linear mixed models (for SBP, DBP, IDWG) and logistic regression models (for RUV) adjusted for DOPPS phase, country, age, sex, Black race, <90 days dialysis vintage, catheter use, BMI, serum albumin, hemoglobin, and 13 comorbidities.
